# Clinical studies evaluating abametapir lotion, 0.74%, for the treatment of head louse infestation

**DOI:** 10.1111/pde.13612

**Published:** 2018-07-12

**Authors:** Vernon M. Bowles, Lisa Jenkins VanLuvanee, Hugh Alsop, Lydie Hazan, Katie Shepherd, Srinivas Sidgiddi, Kent Allenby, Tiina Ahveninen, Sharon Hanegraaf

**Affiliations:** ^1^ Faculty of Veterinary and Agricultural Sciences University of Melbourne Melbourne Vic. Australia; ^2^ Hatchtech Pty Ltd Melbourne Vic. Australia; ^3^ Facet Life Sciences, Inc. Audubon Pennsylvania; ^4^ Axis Clinical Trials Los Angeles California; ^5^ LSRN Research/The Shepherd Institute for Lice Solutions West Palm Beach Florida; ^6^ Promius Pharma a subsidiary of Dr. Reddy's Laboratories Princeton New Jersey

**Keywords:** infestations, therapy‐topical

## Abstract

**Background:**

There is a need for better control of head louse infestations. Abametapir is an inhibitor of metalloproteinases critical for louse survival and egg development. The efficacy of abametapir lotion, 0.74%, was assessed for its ability to clear head louse infestations after a single application.

**Methods:**

Two randomized, double‐blind, multicenter, vehicle‐controlled studies were conducted in subjects aged 6 months and older to compare the effectiveness of abametapir lotion versus vehicle control for eliminating head louse infestations without nit combing. Abametapir lotion was applied to dry hair for 10 minutes on day 0 and then rinsed with water. The primary endpoint was the proportion of index subjects (youngest household member with ≥ 3 live lice at screening) in the intent‐to‐treat population who were louse free at all follow‐up visits through day 14. Older household members with one or more live lice at screening were designated as nonindex subjects and treated as per the index subject within their household.

**Results:**

In the intent‐to‐treat population (index subjects, N = 216), 81.5% of subjects treated with abametapir lotion were louse free through day 14 after a single treatment, versus 49.1% with vehicle (*P* < 0.001). For the combined index and nonindex population (N = 704), 85.9% were louse free through day 14 in the abametapir group, versus 61.3% in the vehicle group (*P* < 0.001). The most frequently reported adverse events were erythema (4.0%), rash (3.2%), and skin burning sensation (2.6%).

**Conclusion:**

Abametapir lotion, 0.74%, was effective at clearing active head louse infestations through day 14 in subjects aged 6 months and older. All adverse events (including one serious but unrelated to study drug) resolved uneventfully.

## 1 INTRODUCTION

Head lice, *Pediculus humanus capitis*, have been infesting human heads for thousands of years.^1^ Louse infestation is estimated to affect 6‐12 million people in the United States annually, primarily children aged 3‐12.^2^ Clinical effects of head louse infestation include excoriation and infection due to scratching, poor sleep, and social disruption.^3^

The majority of head louse infestations are treated with over‐the‐counter (OTC) products containing synergized pyrethrin or synthetic pyrethroid (permethrin), insecticides that target the neurologic system of the adult louse.^3^ However, widespread use of these compounds and their common site of action^4^, ^5^ may be contributing to the development of resistance in lice, resulting in a progressive decline in the effectiveness of these products worldwide.^2^, ^3^ Resistance to pyrethrin and pyrethroid insecticides has been increasing over the last 15 years,^6^, ^7^, ^8^ with recent reports demonstrating that the *kdr‐*resistant allele was detected in 98.3% of 130 sampling sites from 48 U.S. states.^4^ In addition, these agents have little to no direct ovicidal activity,^9^ such that a second application of treatment may be needed to eliminate the lice that hatch from eggs present during the initial treatment. Administration of a second treatment too early, too late, or not at all can result in continued infestation.^10^ This has led to the development of new products based on alternative chemistries and active agents.^11^, ^12^, ^13^, ^14^

Treatments that target lice and their eggs may reduce or eliminate the need for additional treatment applications. New mechanisms of action may be effective against lice that are resistant to pyrethrin or pyrethroids.^8^ Evidence suggests that proteases, including metalloproteinases, are involved in the process of louse egg hatching.^15^ Furthermore, metal‐chelating agents have been shown to inhibit this protease activity in vitro.^15^ Abametapir is a metalloproteinase inhibitor developed to target metalloproteinases critical to the development of adult lice and eggs.^16^

Abametapir lotion is the topical formulation of abametapir being developed for the treatment of head louse infestation.

We report on two identical phase 3 studies comparing the efficacy and safety of a single 10‐minute treatment of abametapir lotion with the efficacy and safety of its vehicle control (an identical formulation without abametapir) in subjects with active head louse infestations.

## 2 SUBJECTS AND METHODS

### 2.1 Study oversight

Two identical phase 3 studies (study 1 and study 2) were conducted as part of the clinical development program for abametapir at multiple U.S. sites. The study design was agreed upon with the Food and Drug Administration under a Special Protocol Assessment. Studies were conducted in compliance with the Declaration of Helsinki, International Conference on Harmonisation guidelines for Good Clinical Practice, and the institutional review board for each investigation site. Written informed consent (parental assent if younger than 18) was obtained from all subjects. Studies were registered at ClinicalTrials.gov: NCT02060903 and NCT02062060.

### 2.2 Study subjects

Eligible subjects were male or female, aged 6 months and older, with an active head louse infestation. Because of the transmittable nature of louse infestations, all family members with head lice were enrolled in these studies. The youngest household member with at least three live head lice was designated as the index subject and included in the primary analysis. Other household members with at least one live head louse were designated as nonindex subjects.

Subjects were excluded if they had used any form of head louse treatment during the 14 days before the study period or an investigational agent during the prior 30 days. Subjects were also excluded if they had visible scalp or skin conditions not attributable to louse infestation (including eczema or atopic dermatitis of the scalp) or had had a prior reaction to any product containing permethrin. If any household member with a louse infestation was not willing to enroll or was ineligible, or a subject was not located in the same household for the study period, all household members were excluded from the study.

### 2.3 Study design

Studies 1 and 2 were randomized, double‐blind, multicenter, vehicle‐controlled, parallel‐group, single‐dose studies. At baseline (day 0), index subjects were randomized to abametapir lotion or vehicle lotion. Nonindex subjects received the same treatment as the index subject within their household. Each subject received a 200‐g bottle of study drug with instructions for application at home by the subject or caregiver on the day of receipt. Product was applied once to the subject's dry scalp and hair and massaged into the scalp and hair, working from the hairline at the back of the neck to the end of the hair. Caregivers were instructed to apply a sufficient amount to achieve saturation, with no more than one bottle of product to be applied regardless of the length and thickness of the hair. Subjects were instructed to cover their face and eyes during application. Once saturation of the hair and scalp was achieved, the product was left on for 10 minutes and then rinsed out with warm water. Nit combing was not permitted for 14 days before and after treatment. Subjects returned to the site and were inspected for the presence of live lice on post‐treatment days 1, 7, and 14. Trained evaluators performed systematic head louse evaluations until live lice were observed or 15 minutes had elapsed. If live lice were detected at any of the three visits, the treatment was considered to have failed, and a commercially available rescue therapy (1% permethrin) was provided. Safety was assessed according to reported adverse events and measurements of vital signs, physical examination, and scalp and eye irritation on days 0, 1, 7, and 14. Scalp irritation included erythema and edema, pruritus, excoriation, and pyoderma. Blood samples for clinical laboratory tests (biochemistry and hematology) were collected on days 0 and 14.

### 2.4 Endpoints

The primary efficacy endpoint was the proportion of index subjects (intent‐to‐treat [ITT]) who were louse free at all follow‐up visits through day 14. Secondary efficacy endpoints were defined as the proportion of index subjects who were louse free at days 1 and 7. An exploratory endpoint was the proportion of all randomized subjects (index and nonindex) who were louse free at all follow‐up visits through day 14. Safety endpoints included adverse events and evaluations for skin, scalp, and ocular irritation.

### 2.5 Statistical analysis

To determine sample size, the expected effect size was estimated based on the results of an earlier phase 2b study and the calculation based on a two‐sided, two‐group, continuity‐corrected, chi‐square test for equal proportion with a 5% level of significance. To detect an effect size that would yield a difference of at least 35% between the vehicle and active treatment groups with 90% power, a sample size of 48 index subjects per group was required. Assuming a 10% dropout rate, an initial enrollment of 53 index subjects (families) per group was required to ensure evaluable data for the primary endpoint from at least 48 index subjects.

The primary and secondary efficacy endpoints for each study were analyzed using the Cochran‐Mantel‐Haenszel test, stratified according to site at a 5% significance level. Interaction between treatment group and site was tested using the Breslow‐Day test; if significant (*P* < 0.10), a logistic regression sensitivity analysis was performed to confirm any treatment site interaction.

## 3 RESULTS

### 3.1 Subjects

**Figure 1:**
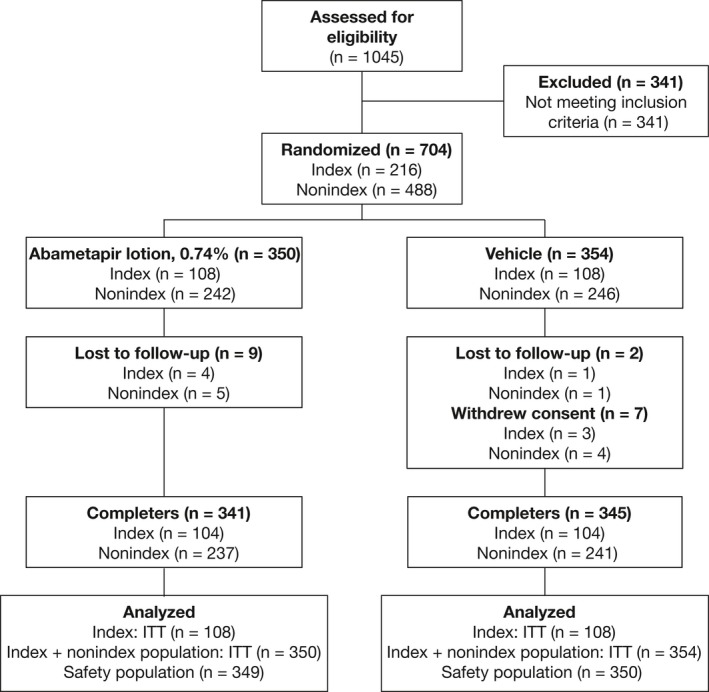
Flowchart of Phase 3 Study Participants' Disposition (study 1 and study 2)

Enrolled subjects ranged in age from 6 months to 61.1 years, with 83.7% females and 16.1% males. The ITT population consisted of 108 index subjects each in study 1 and study 2. Index subjects ranged in age from 6 months to 58.5 years, with 85.2% female and 14.8% male (Table 1). Studies 1 and 2 also included 271 and 217 nonindex subjects (other household members with at least one live head louse), respectively, bringing the total number of randomized subjects to 704 subjects. Overall, 359 subjects were treated with active study drug, 354 subjects were treated with vehicle in the two phase 3 studies, and a total of 686 subjects completed the studies (Figure 1). Demographic characteristics of the ITT population were comparable between treatment groups in both studies. Ethnicity was 80.5% Hispanic for study 1 and 50.2% for study 2; 96% of participants were white. The population included subjects with a range of hair lengths, textures, and thicknesses, which were similar between groups and studies.

**Table 1:** Demographic characteristics of subjects in the intent‐to‐treat populations

Characteristic	Study 1		Study 2	
	Abametapir lotion, 0.74%, n *=* 53	Vehicle lotion, n *=* 55	Abametapir lotion, 0.74%, n *=* 55	Vehicle lotion, n *=* 53
Age, mean ± SD (range)	7.5 ± 4.2 (0.5‐19.2)	7.4 ± 6.7 (1.2‐49.1)	9.8 ± 10.5 (1.6‐58.5)	7.8 ± 7.7 (1.1‐56.9)
Sex, n (%)				
Male	5 (9.4)	10 (18.2)	7 (12.7)	10 (18.9)
Female	48 (90.6)	45 (81.8)	48 (87.3)	43 (81.1)
Weight, kg, mean ± SD (range)	29.2 ± 19.8 (7.5‐125.0)	31.0 ± 22.7 (9.1‐134.5)	33.3 ± 21.1 (10.2‐95.0)	27.8 ± 14.9 (11.0‐83.6)
Height, cm, mean ± SD (range)	116.0 ± 23.7 (67‐165)	117.3 ± 26.9 (53‐163)	125.7 ± 23.4 (76‐173)	120.0 ± 23.9 (71‐170)
Ethnicity, n (%)				
Hispanic	41 (77.4)	46 (83.6)	26 (47.3)	21 (39.6)
Not Hispanic	12 (22.6)	9 (16.4)	29 (52.7)	31 (58.5)
Unknown	0 (0.0)	0 (0.0)	0 (0.0)	1 (1.9)
Race, n (%)				
White	50 (94.3)	55 (100.0)	52 (94.5)	50 (94.3)
Black	2 (3.8)	0 (0.0)	0 (0.0)	2 (3.8)
American Indian or Alaska Native	0 (0.0)	0 (0.0)	1 (1.8)	0 (0.0)
Native Hawaiian or other Pacific Islander	0 (0.0)	0 (0.0)	0 (0.0)	1 (1.9)
Other	1 (1.9)	0 (0.0)	2 (3.6)	0 (0.0)

SD, standard deviation.

### 3.2 Compliance

Individual bottles were returned and weighed to determine product usage. In study 1, subjects randomized to abametapir lotion used a mean amount of 118.5 ± 59.9 g of product, and the vehicle group used 132.8 ± 59.8 g. In study 2, mean usage was 131.2 ± 55.8 g of abametapir lotion and 125.5 ± 55.1 g of vehicle. The amount of product used ranged from 6 g to use of the entire 200 g supplied.

### 3.3 Efficacy

**Figure 2:**
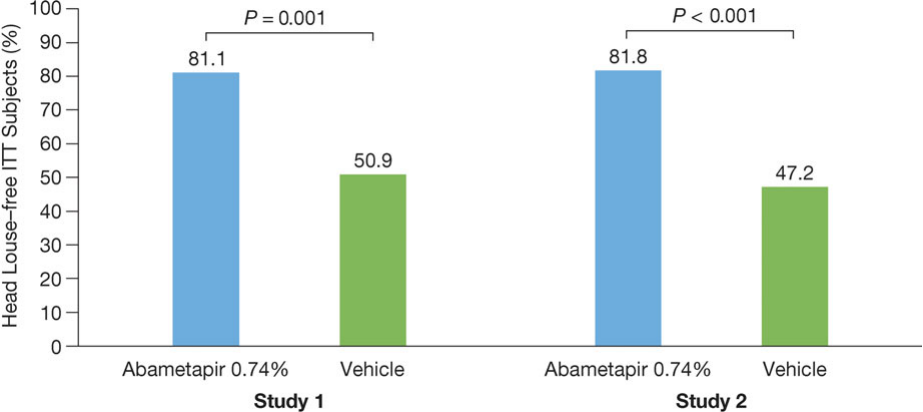
Primary Endpoint results. Percentage of intent‐to‐treat index subjects who were louse free at all visits through day 14 in studies 1 and 2. N* =* 108 for each study. Study 1: abametapir lotion, n* =* 53; vehicle, n* =* 55. Study 2: Abametapir lotion, n* =* 55; vehicle, n* =* 53

The primary efficacy analysis revealed that a significantly greater percentage of index subjects achieved treatment success (no live lice present at any postbaseline visit) with abametapir lotion than with vehicle in study 1 (81.1% vs 50.9%, odds ratio (OR) = 4.01, 95% confidence interval CI: 1.70, 9.48, *P =* 0.001) and study 2 (81.8% vs 47.2%, OR=5.50, 95% CI: 2.20, 13.73, *P* < 0.001) (Figure 2).

**Figure 3:**
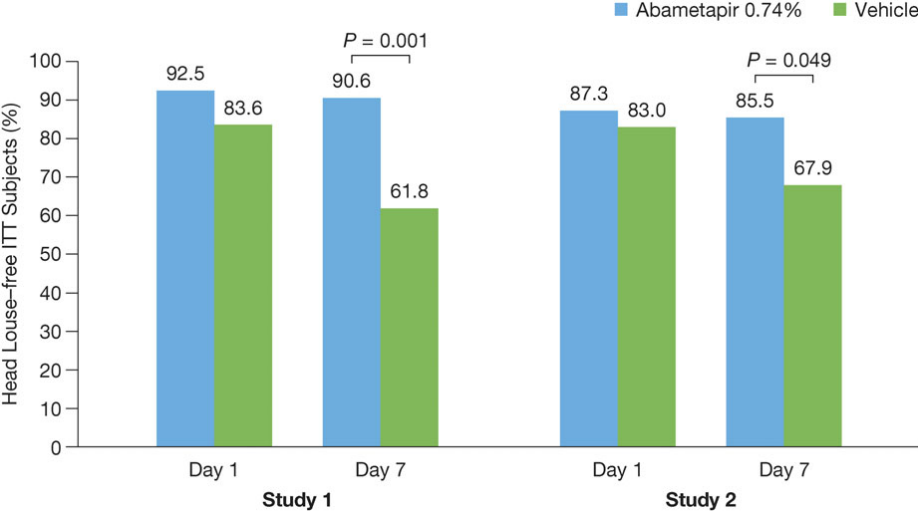
Secondary Endpoints Results. Percentage of intent‐to‐treat index subjects who were louse free at days 1 and 7 in studies 1 and 2. N* =* 108 for each study. Study 1: abametapir lotion, n* =* 53; vehicle, n* =* 55. Study 2: abametapir lotion, n* =* 55; vehicle, n* =* 53

The secondary endpoints for days 1 and 7 indicated that 92.5% of ITT subjects in study 1 and 87.3% in study 2 treated with abametapir lotion were louse free on day 1 after treatment, decreasing slightly to 90.6% in study 1 and 85.5% in study 2 by day 7 after treatment. There was a notable vehicle effect at day 1 that declined to 61.8% by day 7 for study 1 and 67.9% for study 2 (Figure 3).

**Figure 4:**
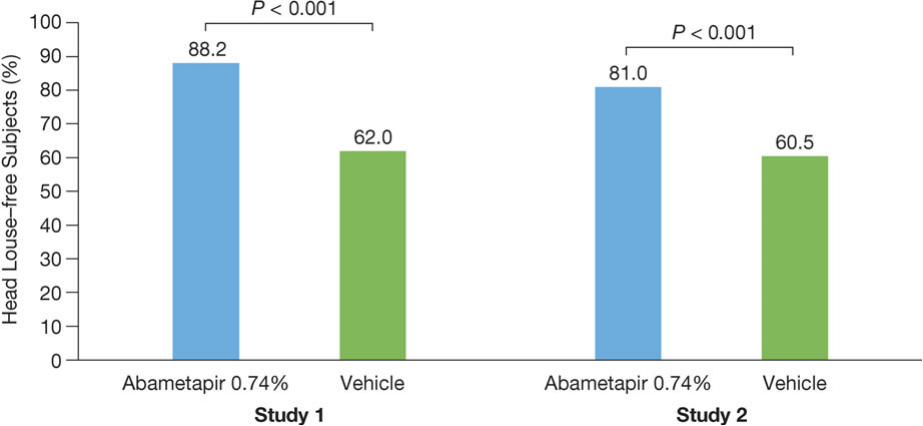
Percentage of all subjects who were louse free at all visits through day 14 in Studies 1 and 2. This population included index and nonindex subjects. N* =* 704. Study 1: abametapir lotion, n* =* 187; vehicle, n* =* 192. Study 2: abametapir lotion n* =* 163; vehicle, n* =* 162

The exploratory endpoint for both studies was to determine the proportion of all randomized subjects who achieved treatment success (were louse free) at all follow‐up visits through day 14. Results for study 1 revealed that 88.2% of the abametapir group and 62.0% of the vehicle group achieved treatment success through day 14 (OR = 4.48, 95% CI: 2.65, 7.60, *P* < 0.001). Similarly, in study 2, 81.0% of the abametapir group and 60.5% of the vehicle group achieved treatment success through day 14 (OR = 2.87, 95% CI: 1.72, 4.78, *P* < 0.001) (Figure 4).

Treatment‐by‐site interaction analyses revealed no significant differences between treatment success and site in study 1. In study 2, a significant site interaction was noted (*P* = 0.096), which was probably driven by a single site having no treatment failures for the active treatment group, although the confirmatory logistic regression sensitivity analysis failed to reveal a significant treatment group‐by‐site interaction for study 2.

### 3.4 Safety

The most frequently reported treatment‐emergent adverse events in the data pooled from studies 1 and 2 were erythema, rash, and skin burning sensation (Table 2). Hair color changes were noted in three subjects from the same site; all resolved by day 7. One serious adverse event was reported in study 1, where a 34‐year‐old woman receiving vehicle was hospitalized for renal impairment during the study period. The adverse event was judged to be unrelated to study treatment. There were no serious adverse events reported in study 2.

**Table 2:** Adverse reactions occurring in at least 1% of the abametapir lotion, 0.74% group and at a greater frequency than in the vehicle group (studies 1 and 2)

Adverse reactions	Abametapir lotion, 0.74%, N = 349	Vehicle lotion N = 350
	n (%)	n (%)
Erythema	14 (4.0)	6 (1.7)
Rash	11 (3.2)	8 (2.3)
Skin burning sensation	9 (2.6)	0 (0.0)
Contact dermatitis	6 (1.7)	4 (1.1)
Vomiting	6 (1.7)	2 (0.6)
Eye irritation	4 (1.2)	2 (0.6)
Hair color changes	3 (1.0)	0 (0.0)

Hematology and biochemistry analysis showed no trends or clinically meaningful changes after administration of abametapir lotion or vehicle lotion. All analyses indicated that abametapir lotion was safe and well tolerated.

## 4 DISCUSSION

Abametapir is an agent being developed for the treatment of head louse infestations. In two large phase 3 studies conducted in a total of 704 subjects aged 6 months and older, abametapir lotion eliminated lice in more than 80% of subjects after a single 10‐minute application with no nit combing. Abametapir demonstrated significantly greater efficacy in the clearance of head louse infestations than vehicle, with comparable results seen between the two studies.

The life cycle of the louse ranges from 33 to 35 days and includes egg, nymph, and adult stages. If a product cannot kill louse eggs through chemical or physical action, a second treatment may be necessary to eliminate nymphs emerging from unaffected eggs. In the two studies presented here, 81.1% and 81.8% of ITT subjects were louse free at day 14 after a single treatment. These data indicate that abametapir kills lice, and by inference also their eggs. In a previously completed in vitro study, topical contact with abametapir lotion showed 100% efficacy against head louse eggs.^16^

The vehicle used in the abametapir lotion resulted in 47.2% and 50.9% of louse‐free subjects at day 14. Some degree of vehicle effect was anticipated in the clinical studies, as an in vitro study of our vehicle formulation reported that it can provide up to 55% ovicidal efficacy against head lice compared with 10–20% for the water control. 16 It is currently not clear as to what component or components of the vehicle formulation may be contributing to this observed vehicle effect.

The incidence of adverse events was low and consistent with a topical product, with the most common adverse events being erythema, rash, and skin burning sensation. There were no discontinuations associated with abametapir lotion in any age group. Hair color changes were noted in three subjects. The abametapir compound can chelate iron at concentrations as low as 1 part per million. High iron content in the water used to rinse the product from the hair may have resulted in the observed color changes, which resolved within 7 days.

One limitation of these studies is that reinfestation was not specifically assessed or quantified. To eliminate any possibility of reinfestation, subjects would need to be isolated for the 2 weeks after treatment, a methodology that would present significant practical and ethical obstacles in the population studied here.

## 5 CONCLUSION

Abametapir lotion, 0.74%, was effective for the topical treatment of head louse infestation in subjects aged 6 months and older with a single administration. The incidence of adverse events was low, with the most common adverse events being erythema, rash, and skin burning sensation. The inhibition of metal‐dependent processes, including metalloproteinases, in adult lice and lice eggs, is a novel mechanism of action that head louse treatments have not previously used.

## CONFLICT OF INTEREST

Vernon Bowles and Hugh Alsop are stockholders in Hatchtech Pty Ltd. Srinivas Sidgiddi is an employee of Dr. Reddy's Laboratories and owns stock in the company. Kent Allenby was an employee of Dr. Reddy's Laboratories until August 2017 and owns stock in the company. The other authors have no potential conflicts of interest relevant to this article to disclose.
